# Methylation-dependent MCM6 repression induced by LINC00472 inhibits triple-negative breast cancer metastasis by disturbing the MEK/ERK signaling pathway

**DOI:** 10.18632/aging.103568

**Published:** 2021-02-26

**Authors:** Guoli Shao, Xulong Fan, Pusheng Zhang, Xuewen Liu, Lei Huang, Shufeng Ji

**Affiliations:** 1 Special Medical Service Center, Zhujiang Hospital of Southern Medical University, Guangzhou 510280, P. R. China; 2 Department Breast Surgery, Maternity and Children’s Healthcare Hospital of Foshan, Foshan 528000, P. R. China; 3 Department General Surgery, Zhujiang Hospital of Southern Medical University, Guangzhou 510280, P. R. China

**Keywords:** long non-coding RNA, LINC00472, riple-negative breast cancer, metastasis, minichromosome maintenance complex component 6

## Abstract

Long noncoding RNAs (lncRNAs) have been identified to be dysregulated in multiple cancer types, which are speculated to be of vital significance in regulating several hallmarks of cancer biology. Triple-negative breast cancer (TNBC) is acknowledged as an aggressive subtype of breast cancer. In this study, we found the lncRNA LINC00472 was poorly expressed in TNBC tissues and cells. Overexpression of LINC00472 could inhibit the proliferation, invasion and migration of MDA-MB-231 cells. On the contrary, minichromosome maintenance complex component 6 (MCM6) was highly expressed in TNBC tissues and MDA-MB-231 cells due to suppressed methylation. LINC00472 induced site-specific DNA methylation and reduced the MCM6 expression by recruiting DNA methyltransferases into the MCM6 promoter. Since the restoration of MCM6 weakened the tumor-suppressive effect of LINC00472 on MDA-MB-231 cells, LINC00472 potentially acted as a tumor suppressor by inhibiting MCM6. In addition, *in vivo* experiments further substantiated that overexpression of LINC00472 inhibited tumor growth and metastasis to lungs by decreasing the expression of MCM6. Overall, the present study demonstrated that LINC00472-mediated epigenetic silencing of MCM6 contributes to the prevention of tumorigenesis and metastasis in TNBC, providing an exquisite therapeutic target for TNBC.

## INTRODUCTION

Triple-negative breast cancer (TNBC), accounting for 15% incidence among all types of BC, is characterized by radical loss of progesterone receptors and estrogen receptors expression as well as absence of overexpression of amplification of the human epidermal growth factor receptor-2, the 3 typical biomarkers for BC [1]. As a heterogeneous disorder, TNBC possesses as a threat to patients with undesirable oncologic outcomes and limited therapeutic modalities with chemotherapy prevailing as the primary therapeutic preference [2, 3]. In recent years, substantial literature has provided comprehensive findings eliciting the implications of long noncoding RNAs (lncRNAs) in the diagnosis and therapies for cancer [4]. Notably, the oncogenic function of several lncRNAs in BC has been identified with regard to their effects on cell proliferation and apoptosis in BC [5]. For instance, LINC00472 has been recognized to serve as a tumor suppressor in BC in association with the survival time of patients [6, 7]. Interestingly, research has elicited a relation between the function of LINC00472 in BC and the estrogen receptor-α, a vital biomarker for prognosis [8]. Additionally, several lncRNAs have been documented to participate in the initiation and progression of TNBC [9]. Therefore, from the aforementioned literature, it is inferred that LINC00472 may also be involved in TNBC, hereby, making it a viable target for further investigation.

Minichromosome maintenance complex components (MCMs), consisting of MCM2 - MCM10, are defined as critical diagnostic and prognostic markers, while MCM6 serves as a comprehensive indicator of the poor survival condition of patients with glioma [10]. MCM6 pertaining as the component for the putative replicative helicase MCM2-7 complex is crucial for the initiation and elongation of DNA replication [11, 12]. For instance, the participation of MCM6 in the cell-cycle progression has been reported in BC cells [13]. More recently, a study documented a high expression of MCM6 in TNBC [14], but whether MCM6 can affect cancer progression and metastasis in this malignancy is uncertain. Considering the negative correlation between LINC00472 and MCM6 in TNBC on the basis of microarray-based expression analysis prior to our study, we intended to investigate the functional significance of this interaction in TNBC. Essentially, an existing study elucidated the significance of the mitogen-activated protein kinases/extracellular signal-regulated kinase (MEK/ERK) signaling pathway in respect to the therapeutic value of MCM6 in hepatocellular carcinoma [15]. The MEK/ERK signaling pathway has been validated to provide promising targets for developing efficacious drugs against cancers due to its regulatory role in cell survival during different stages of cancer [16]. Studies have elicited the functionality of the MEK/ERK signaling pathway in TNBC [17, 18]. Therefore, we aim to verify the involvement of LINC00472 through its interaction with MCM6 *via* the MEK/ERK signaling pathway in TNBC. It is hoped that the question will be addressed with our proposed approach as follows.

## RESULTS

### LINC00472 was under-expressed in TNBC tissues and human TNBC cell lines

**Figure 1:**
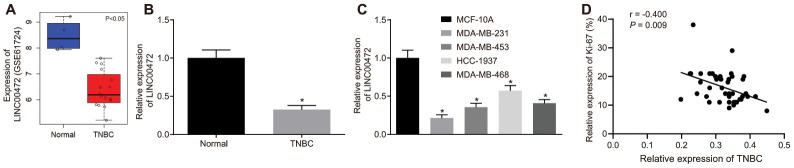
**TNBC tissues and cells exhibit low expression of LINC00472 and the expression of LINC00472 is negatively correlated with Ki-67 expression.** (**A**) The expression of LINC00472 in normal and TNBC tissues in microarray dataset GSE61724, blue referring to normal tissues and red referring to TNBC tissues. (**B**) The expression of LINC00472 in normal and TNBC tissues normalized to GAPDH determined by RT-qPCR (n = 42). (**C**) The expression of LINC00472 in normal and TNBC cell lines normalized to GAPDH determined by RT-qPCR. (**D**) The correlation between LINC00472 and Ki-67 analyzed by Pearson’s correlation coefficient. * *p* < 0.05 *vs.* the normal tissue or the MCF-10A cell line. The results were measurement data and expressed as mean ± standard deviation. Data comparison in (**B**) was analyzed by paired *t*-test and in (**C**) was analyzed by one-way ANOVA, followed by Tukey’s post hoc test. The experiment was conducted 3 times independently.

The expression profiles of GSE61724 and the relevant probe annotation (novel transcripts associated with lymph node metastasis in TNBC) were downloaded from the Gene Expression Omnibus (GEO) database (https://www.ncbi.nlm.nih.gov/geo/). Those profiles were analyzed using the limma package of R language (http://master.bioconductor.org/packages/release/bioc/html/limma.html) so as to determine the differentially expressed transcripts in compliance with the threshold as |logFoldChange| > 2 and *p* value < 0.05. Microarray data analysis of GSE61724 showed that lncRNA LINC00472 was a downregulated in context of TNBC (Figure 1A). To identify the expression profile of LINC00472 in TNBC, RT-qPCR was performed to determine the expression of LINC00472 in TNBC tissues and cells. Results revealed that the LINC00472 expression was significantly lower in TNBC tissues compared to the normal breast tissues (*p* < 0.05, Figure 1B). Likewise, the TNBC cell lines (MDA-MB-231, MDA-MB-453, HCC-1937 and MDA-MB-468) exhibited significantly lower LINC00472 expression relative to the human normal breast cell line (MCF-10A), among which the lowest LINC00472 expression was detected in the MDA-MB-231 cell line (*p* < 0.05, Figure 1C). Therefore, the MDA-MB-231 cell line was selected for subsequent experimentation.

Considering the aberrant expression of LINC00472 in TNBC tissues, an analysis was conducted based on the correlation between the LINC00472 expression and the clinicopathological characteristics in the 42 cases of TNBC (Table 1). Results demonstrated significant correlations between LINC00472 and histological grading, lymph node metastasis, clinical grading and the Ki-67 expression (*p* < 0.05). However, no significant correlation was identified between LINC00472 and patient age (*p* > 0.05). The correlation between LINC00472 expression and Ki-67 expression was further analyzed using the Pearson’s correlation coefficient, which suggested the presence of an intimate correlation between them (r^2^ = 0.9588) (Figure 1D). Conjointly, these findings indicated that LINC00472 is poorly expressed in TNBC, which is associated to the development and metastasis of TNBC.

**Table 1:** The association between LINC00472 and clinicopathological characteristics of patients with triple-negative breast cancer.

**Clinicopathological characteristics**	**n**	**LINC00472 expression**		
		**Low expression**	**High expression**	***p***
		**(n = 18)**	**(n = 24)**	
Age (year)				0.367
> 60	22	11	11	
≤ 60	20	7	13	
Tissue differentiation				0.015
Poor	22	14	8	
Moderate	17	3	14	
Well	3	1	2	
Clinical grading				0.013
Ι + II	19	4	15	
III	23	14	9	
Lymph node metastasis				0.012
No	24	6	18	
Yes	18	12	6	
Ki-67 (%)				0.011
< 15 %	22	5	17	
≥ 15 %	20	13	7	

Note: The data were measurement data, expressed as the number of cases and tested by chi-square test. *p* < 0.05 was indicative of significant difference.

### Overexpression of LINC00472 inhibited MDA-MB-231 cell proliferation, migration and invasion

**Figure 2:**
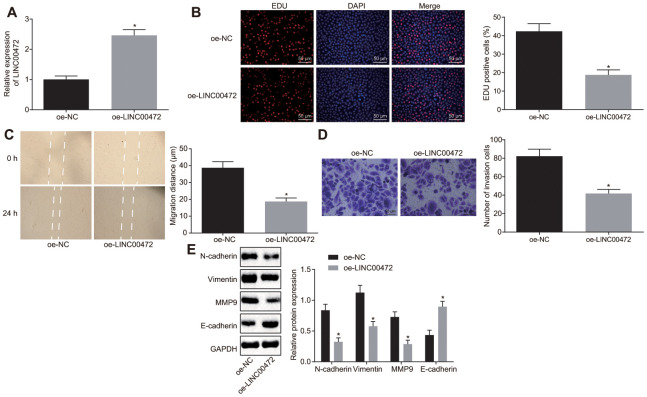
**Overexpressed LINC00472 suppresses TNBC cell proliferation, migration and invasion.** (**A**) The expression of LINC00472 in MDA-MB-231 cells normalized to GAPDH as determined by means of RT-qPCR. (**B**) MDA-MB-231 cell proliferation detected by EdU assay(× 200). (**C**) MDA-MB-231 cell migration detected by scratch test. (**D**) MDA-MB-231 cell invasion detected by Transwell assay (× 200). (**E**) The protein expression of N-cadherin, Vimentin, MMP9 and E-cadherin normalized to GAPDH determined by Western blot analysis. * *p* < 0.05 *vs.* the oe-NC group (MDA-MB-231 cells transfected with oe-NC). The results were measurement data and expressed as mean ± standard deviation. Data comparison between two groups was analyzed by independent sample *t*-test. The experiment was conducted 3 times independently.

With results determining the correlation of LINC00472 with TNBC development, the focus was shifted on analyzing the effects of LINC00472 on TNBC cellular behaviors, which were evaluated by means of EdU, scratch and Transwell assays, respectively. The expression of LINC00472 was detected by means of RT-qPCR. The obtained data exhibited that oe-LINC00472 delivery induced a significantly elevated LINC00472 expression (*p* < 0.05, Figure 2A), suppressed cell proliferation (*p* < 0.05, Figure 2B), migration (*p* < 0.05, Figure 2C) and invasion (*p* < 0.05, Figure 2D). Western blot analysis was conducted to measure the expression of several epithelial-mesenchymal transition (EMT) markers N-cadherin, Vimentin, E-cadherin and metastatic-related marker MMP9, results of which showed that the expression of N-cadherin, Vimentin, and MMP9 decreased significantly while the E-cadherin expression increased essentially in the presence of oe-LINC00472 (*p* < 0.05, Figure 2E). To conclude, overexpressed LINC00472 expression is appropriate to inhibit the proliferation, migration and invasion of TNBC cells.

### LINC00472 mediated methylation-dependent MCM6 inhibition in MDA-MB-231 cells

**Figure 3:**
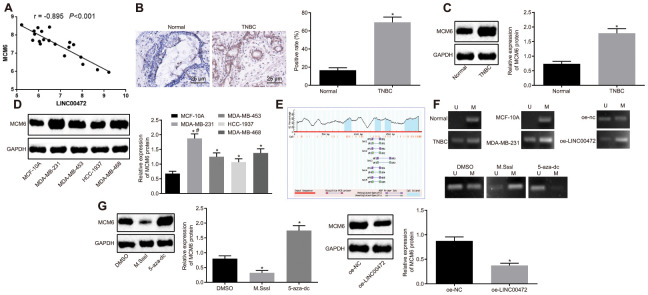
**LINC00472 increases MCM6 promoter methylation to inhibit the MCM6 expression.** (**A**) A negative correlation between LINC00472 and MCM6 in the microarray dataset GSE61724. (**B**) The expression of MCM6 in TNBC tissues normalized to GAPDH identified by means of immunohistochemistry (× 400), * *p* < 0.05 *vs.* the normal tissues. (**C**) The expression of MCM6 in TNBC tissues normalized to GAPDH as determined by means of Western blot analysis, * *p* < 0.05 *vs.* the normal tissues. (**D**) The expression of MCM6 in TNBC cell lines normalized to GAPDH determined by means of Western blot analysis, * *p* < 0.05 *vs.* the MCF-10A cell line, # *p* < 0.05 *vs.* the HCC-1937 or MDA-MB-468 cell line. (**E**) The CpG island in the MCM6 promoter region predicted on MethPrimer website (https://www.urogene.org). (**F**) The methylation in the MCM6 promoter region as detected by means of MSP assay. (**G**) The expression of MCM6 in MDA-MB-231 cells normalized to GAPDH after treatment with M.SssI or 5-aza-dc or after transfection with oe-LINC00472 determined by means of Western blot analysis, * *p* < 0.05 *vs.* the DMSO (MDA-MB-231 cells treated with DMSO) or oe-NC group (MDA-MB-231 cells transfected with oe-NC). The results were measurement data and expressed as mean ± standard deviation. Data comparison in (**B** and **C**) was analyzed by the paired *t*-test, in (**D**) was analyzed by one-way ANOVA followed by Tukey’s post hoc test and in (**G**) was analyzed by the unpaired *t*-test. The experiment was conducted 3 times independently.

Additionally, the microarray data analysis of GSE61724 revealed a negative correlation between LINC00472 and MCM6 (Figure 3A). For verification purposes, the expression of MCM6 was measured in TNBC tissues by means of immunohistochemistry and Western blot analysis, results of which confirmed the presence of a notably higher MCM expression in TNBC tissues compared to the normal breast tissues (*p* < 0.05, Figure 3B, 3C). The protein determination conducted in the TNBC cell lines by Western blot analysis showed that the TNBC cell lines (MDA-MB-231, MDA-MB-453, HCC-1937 and MDA-MB-468) exhibited a significantly higher MCM6 expression compared to the human normal breast cell line (MCF-10A), among which the highest MCM6 expression was detected in the MDA-MB-231 cell line (*p* < 0.05, Figure 3D). The, MethPrimer website (https://www.urogene.org) was adopted for prediction of the CpG island in the MCM6 promoter region. The obtained results supported the presence of CpG island in the MCM6 promoter region (Figure 3E). The methylation levels in the TNBC tissues and cells were determined by conducting MSP assay. Results revealed evident methylation at a specific site in the normal and MCF-10A groups while partial methylation was detected in the TNBC and MDA-MB-231 groups. Subsequently, the MDA-MB-231 cells were treated with DMSO, CpG methyltransferase, M.SssI (EM0821; Thermo Fisher Scientific, Waltham, MA, USA), or inhibitor of DNA methyltransferase, 5-Aza-2’-deoxycytidine (5-aza-dc; #N4003; New England Biolabs, Ipswich, MA, USA) for 1 h or transfected with oe-NC or oe-LINC00472. After the aforementioned treatments, the site-specific methylation in the MCM6 promoter was detected. Results revealed partial site-specific methylation in the presence of DMSO or oe-NC, methylation in the presence of M.SssI or oe-LINC00472 and no methylation in the presence of 5-aza-dc (Figure 3F). The expression of MCM6 was further determined by means of Western blot analysis. Results showed that the MCM6 expression was lowered by M.SssI and elevated by 5-aza-dc in comparison to the DMSO treatment, and that was reduced by oe-LINC00472 in comparison to oe-NC (*p* < 0.05, Figure 3G). The afore-mentioned findings suggest that LINC00472 induces methylation of the MCM promoter to inhibit the MCM expression profile.

### LINC00472 diminished the expression MCM6 by recruiting DNMT1, DNMT3a and DNMT3b into the MCM6 promoter in MDA-MB-231 cells

**Figure 4:**
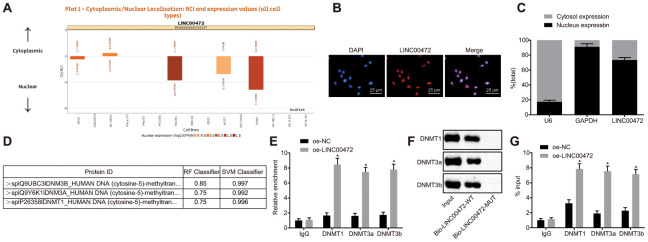
**LINC00472 down-regulates MCM6 expression via recruitment of DNA methyltransferases.** (**A**) The subcellular localization of LINC00472 predicted on lncATLAS. (**B**) The subcellular localization (× 400) of LINC00472 detected by FISH assay. (**C**) The nuclear and cytoplasmic expression of LINC00472 in MDA-MB-231 cells as determined by means of RT-qPCR. (**D**) The binding relation between LINC00472 and DNMT1, DNMT3a or DNMT3b analyzed on RPISeq, with Random Forest and Support Vector Machine > 0.5 indicative of positive results. (**E**) The binding relation between LINC00472 and DNMT1, DNMT3a or DNMT3b relative to IgG detected by means of RIP assay. (**F**) The expression of DNMT1, DNMT3a or DNMT3b pulled down by LINC00472 normalized to Input. (**G**) The enrichment of DNMT1, DNMT3a and DNMT3b in MCM6 promoter region normalized to IgG detected by means of ChIP assay. * *p* < 0.05 *vs.* the oe-NC group (MDA-MB-231 cells treated with oe-NC). The results were measurement data and expressed as mean ± standard deviation. Data comparison between two groups was analyzed by the independent sample *t*-test. The experiment was conducted 3 times independently.

According to the results on lncATLAS (http://lncatlas.crg.eu/), LINC00472 was predominantly localized in the nucleus (Figure 4A), which was further verified by subsequent regimens of FISH and subcellular fractionation location assays (Figure 4B–4C). The prediction results obtained from RPISeq (http://pridb.gdcb.iastate.edu/RPISeq/) revealed that LINC00472 might bind to DNMT1, DNMT3a and DNMT3b (Figure 4D). RIP and RNA pull-down assays were performed for further verification of the same. Results showed that compared to the oe-NC group, the enrichment of DNMT1, DNMT3a and DNMT3b was significantly elevated by oe-LINC00472 (*p* < 0.05, Figure 4E). Besides, Bio-LINC00472-WT could pull down DNMT1, DNMT3a and DNMT3b while Bio-LINC00472-MUT was incapable of doing the same (Figure 4F), suggesting that LINC00472 could recruit DNMT1, DNMT3a and DNMT3b. The website analysis by LongTarget tool revealed complementary base pairing of LINC00472 with the MCM6 promoter in the form of RNA-DNA. The enrichment of DNMT1, DNMT3a and DNMT3b in the MCM6 promoter region was further detected by means of ChIP assay, which demonstrated that oe-LINC00472 induced more enrichment of DNMT1, DNMT3a and DNMT3b in comparison to oe-NC (*p* < 0.05, Figure 4G). The afore-mentioned findings demonstrate that LINC00472 recruits DNMT1, DNMT3a and DNMT3b to the MCM6 promoter region to mediate methylation of the MCM6 promoter region.

### MCM6 activated the MEK/ERK signaling pathway and LINC00472 enhanced TNBC progression *in vitro* by inhibiting MCM6

**Figure 5:**
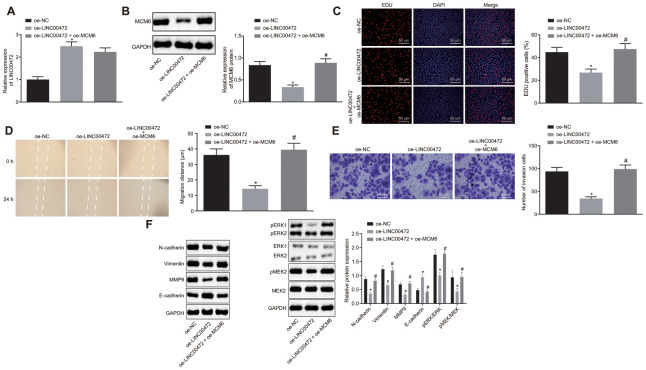
**Overexpression of MCM6 activated MEK/ERK signaling pathway and restored the proliferation, migration and invasion of TNBC cells inhibited by LINC00472.** (**A**) The expression of LINC00472 in the MDA-MB-231 cells normalized to GAPDH as determined by means of RT-qPCR. (**B**) The expression of MCM6 in MDA-MB-231 cells normalized to GAPDH determined by means of Western blot analysis. (**C**) MDA-MB-231 cell proliferation detected by EdU assay (× 200). (**D**) MDA-MB-231 cell migration detected by scratch test. (**E**) MDA-MB-231 cell invasion detected by Transwell assay (× 200). (**F**) The protein expression of N-cadherin, Vimentin, MMP9, E-cadherin, p-ERK, ERK, p-MEK and MEK normalized to GAPDH determined by means of Western blot analysis. * *p* < 0.05 *vs.* the oe-NC group (MDA-MB-231 cells treated with oe-NC). # *p* < 0.05 *vs.* the oe-LINC00472 group (MDA-MB-231 cells treated with oe-LINC00472). The results were measurement data and expressed as mean ± standard deviation. Data comparison among multiple groups was analyzed by one-way ANOVA followed by the Tukey’s post hoc test. The experiment was conducted 3 times independently.

To study the effects of LINC00472/MCM6 on TNBC cell proliferation, migration and invasion, we ventured to up-regulate LINC00472 and silence MCM6 in the MDA-MB-231 cells. RT-qPCR was conducted to assess the LINC00472 expression and the results showed that the provision of oe-LINC00472 solitary significantly up-regulated LINC00472 expression and down-regulated MCM6 expression (*p* < 0.05), while the provision of oe-MCM6 restored the MCM6 expression (*p* > 0.05, Figure 5A–5B). The TNBC cell proliferation, migration and invasion were evaluated by means of EdU assay, scratch test and Transwell assay, respectively. Results showed that cells transduced with oe-LINC00472 exhibited evidently suppressed cell proliferation (Figure 5C), migration (Figure 5D) and invasion (Figure 5E), all of which could were found to be reversed by restoration of MCM6 (*p* < 0.05). Western blot analysis was performed to measure the protein levels of the metastatic markers and the MEK/ERK signaling pathway-related genes. Results revealed that the delivery of oe-LINC00472 individually resulted in lower expression of N-cadherin, Vimentin and MMP9, p-ERK/ERK and p-MEK/MEK ratios but a higher E-cadherin expression (*p* < 0.05). The elevated expression of N-cadherin, Vimentin and MMP9, p-ERK/ERK and p-MEK/MEK ratios and reduced E-cadherin expression were elevated by oe- MCM6 in the presence of oe-LINC00472 (*p* < 0.05, Figure 5F). Conjointly, overexpressed LINC00472 can suppress proliferation, migration and invasion of TNBC cells by inhibiting the MCM6 expression via inactivation of the MEK/ERK signaling pathway.

### Overexpression of LINC00472 inhibited tumor growth and lung metastasis *in vivo* through downregulation of MCM6

**Figure 6:**
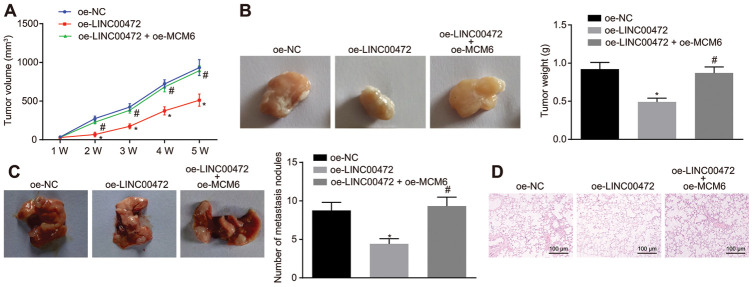
**Overexpression of LINC00472 inhibited tumor growth and metastasis to lungs *in vivo* through downregulation of MCM6.** (**A**) The tumor volume of nude mice (n = 12). (**B**) The representative images of resected tumors and the tumor weights (n = 12). (**C**) The representative images and number of metastasis nodules in the mice (n = 12). (**D**) The pathological changes in lung tissues identified by means of HE staining (× 100). * *p* < 0.05 *vs.* the oe-NC group (nude mice bearing MDA-MB-231 cells treated with oe-NC). # *p* < 0.05 *vs.* the oe-LINC00472 group (nude mice bearing MDA-MB-231 cells treated with oe-LINC00472). The results were measurement data and expressed as mean ± standard deviation. Data comparison among multiple groups was analyzed by one-way ANOVA followed by the Tukey’s post hoc test. Data comparison at different time points was analyzed by repeated measures ANOVA, followed by Bonferroni’s post hoc test. n = 12. The experiment was conducted 3 times independently.

Lastly, the *in vivo* effects of LINC00472/MCM6 on TNBC cell growth were explored by inoculating the stably transfected MDA-MB-231 cells into the right axilla of nude mice for tumor formation. The analysis on tumor growth, size and weight revealed that the LINC00472 overexpression resulted in smaller tumor size and weight, both of which were increased by upregulation of MCM6 (*p* < 0.05, Figure 6A, 6B). Further investigative attempts corresponding to the tumor metastasis *in vivo* revealed the presence of fewer metastasis nodules in mice injected with oe-LINC00472-transfected MDA-MB-231 cells. However, a higher proportion of metastasis nodules in mice were induced by MCM6 overexpression in the presence of LINC00472 (*p* < 0.05, Figure 6C). Besides, HE staining of the lung tissues showed that the extent of lesion was milder after overexpression of LINC00472, however severe lesions were observed after up-regulation of both LINC00472 and MCM6 in contrast to up-regulation of MCM6 individually (*p* < 0.05, Figure 6D). The evidence suggests that the inhibitory effects of overexpressed LINC00472 are valid on tumor growth and metastasis to lungs *in vivo*, which could be induced by inhibition of MCM6.

## DISCUSSION

BC is well acknowledged as the most prevalent cause of neoplasia and mortality within the female population across the world [19]. Thereinto, TNBC remains as an excruciating challenge in spite of novel developments made for improvement of the therapeutic approaches in recent years [20]. Recent researches have highlighted the notable advancements in understanding the carcinogenesis mechanism and anti-cancer drug development [21–24]. Interestingly, lncRNAs have gained attention as regulators of BC tumorigenesis due to interactions with other functional molecules [25, 26]. The investigative attempts were hereby made in the current study to unravel the role of LINC00472 in TNBC through interaction with MCM6 and the MEK/ERK signaling pathway. The available evidence conferred that overexpressed LINC00472 leads to suppression of the TNBC cell oncogenic properties *in vitro* as well as inhibited tumor growth *in vivo* by down-regulating MCM6 and blocking the MEK/ERK signaling pathway.

The chief finding of our study revealed poorly expressed LINC00472 and highly expressed MCM6 in TNBC tissues and cells. Furthermore, a series of rescue experiments were designed and performed with results highlighting the capability of overexpressed LINC00472 to function as a tumor suppressor due to inhibition of TNBC cell proliferation, migration and invasion as well as tumor growth and metastasis. The down-regulation of LINC00472 has been detected in colorectal cancer tissues and cells as well while overexpressed LINC00472 inhibits colorectal cancer cell proliferation and tumor growth and promotes cell apoptosis [27]. Moreover, a low expression of LINC00472 has demonstrated association with the metastasis of patients with hepatocellular carcinoma and overexpressed LINC00472 leads to inhibited cell proliferation, migration and invasion along with enhanced cell apoptosis [28]. In consistency with our results, similar tumor-suppressive functionality of LINC00472 has been validated in research based on lung adenocarcinoma [29]. Besides, accumulative evidence has elicited the suppressive effects induced by LINC00472 particularly in BC [6, 7].

As an initiator of DNA replication up-regulated in the G0 phase, MCM6 has been identified to serve as a biomarker for undesirable clinical outcomes of patients with endometrial cancer and glioma [10, 30]. Studies have documented the ectopic expression of MCM6 in TNBC [14], whereas, whether silencing MCM6 could reverse the unfavorable circumstances in TNBC still requires investigation. Essentially, the promoter methylation and mutation of certain tumor suppressors or oncogenes have gained recognition with regard to tumorigenesis [31–33], thereby speculating the involvement of methylation and expression of MCM6 in TNBC. Subsequent mechanistic analyses of our study revealed a negative correlation between LINC00472 and MCM6 where MCM6 could be down-regulated by LINC00472 by inducing MCM6 promoter methylation by incorporating DNMT1, DNMT3a and DNMT3b to the MCM6 promoter region. Over a series of gain-of-function and rescue experiments, the results revealed that the methylation-dependent repression of MCM6 due to LINC00472 mediation was the potential mechanism substantiating the anti-tumor and anti-metastatic properties of LINC00472 in TNBC.

Additionally, our findings demonstrated the involvement of the MEK/ERK signaling pathway as a radical modulator of LINC00472/MCM6 in TNBC progression. The MEK/ERK signaling pathway was hyper-activated in approximately 50% of TNBC corresponding to the genomic loss of dual specificity phosphatase 4, a mediator of ERK1/2 in a negative manner [17]. Our findings revealed the significantly diminished p-ERK/ERK and p-MEK/MEK ratios in response to overexpressed LINC00472, suggesting that LINC00472 impeded activation of the MEK/ERK signaling pathway in TNBC. Also, restoration of MCM6 reversed the inhibitory effect of LINC00472 on MEK/ERK signaling pathway, which was consistent with the similar regulatory mechanism in hepatocellular carcinoma that activation of the MEK/ERK signaling pathway has been found to be associated with the promotion of metastasis induced by MCM6 [15]. This highlighted the potential involvement of MEK/ERK signaling pathway in TNBC metastasis under LINC00472/MCM6 mediation. However, LINC00472 may function as a mediator for various signaling pathways in different human cancers. For instance, LINC00472 impedes the PTEN/PI3K/AKT signaling pathway so as to restrain bladder tumor growth and metastasis [34], while concurrently activating the p53 signaling pathway to retard the progression of non-small-cell lung cancer [35]. Whether other pathways may be regulated by LINC00472 in TNBC warrants further exploration.

To conclude, the present study provides evidence eliciting that overexpression of LINC00472 can suppress cancer progression and metastasis *via* down-regulation of MCM6 in TNBC. Our study serves as an insight for developing novel anti-tumor and anti-metastatic regimens for TNBC. However, the metastatic routes of TNBC include multiple organs and multiple systems. Future studies are warranted for investigating the other metastatic routes apart from lung metastasis based on this investigation. Besides, studies should focus on documenting and treating the possible side effects (e.g., impacts on proliferation and renewal of normal tissues) induced by MCM6 in the future.

## MATERIALS AND METHODS

### Clinical sample collection

Forty-two paired tumor tissues and adjacent non-tumor tissues were surgically resected from 42 female patients (mean age: 55.98 ± 17.01 years) who were diagnosed with TNBC by pathological examination and magnetic resonance-diffusion weighted imaging (MR-DWI) in the Zhujiang Hospital of Southern Medical University from April 2015 to February 2017. The inclusion criteria for the patients were as follows: (1) they were pathologically confirmed with TNBC by surgical resection with complete clinical data; (2) no prior history of breast enhancement, mastitis, radiochemotherapy or other breast-related surgeries; and (3) voluntary participation with signed informed consents. The exclusion criteria for the patients were as follows: (1) no pathologically confirmation of TNBC; (2) they underwent MR-DWI at least 1 month after the surgery; (3) reports of cancer recurrence and distant metastasis after treatment; (4) prior history of psychiatric disorders and inability to finish questionnaire. Besides, the pregnant or lactating patients were excluded. The sample collection was performed in strict accordance with the guidelines. A portion of the sample was stored at -80°C while the remaining portion was fixed using 10% formalin, dehydrated and embedded with paraffin. The study was conducted with approval of the Institutional Review Board of Zhujiang Hospital of Southern Medical University. All participants provided written informed consent prior to participation.

### Immunohistochemistry for MCM6 detection

The paraffin-embedded tissue sample was divided into 5 μm sections. A section blockade was conducted using the normal goat serum (C-0005, Shanghai Haoran Biological Technology Co., Ltd., Shanghai, China) at room temperature for 20 min and then incubated with the primary rabbit anti-human antibody to MCM6 (ab190948, 1 : 500, Abcam Inc., Cambridge, UK) at 4°C overnight, and with the secondary goat anti-rabbit antibody to Immunoglobulin G (IgG) (ab6785, 1 : 1000, Abcam Inc.) at 37°C for 20 min and with the horseradish peroxidase (HRP)-conjugated streptavidin protein working solution (0343-10000U, Imunbio Co., Ltd., Beijing, China) at 37°C for 20 min. Then, 3’3-diaminobezidin (ST033, Whiga Biotechnology Co., Ltd., Guangzhou, Guangdong, China) was supplemented to the sections for development. Sections were than counter-stained using hematoxylin (PT001, Shanghai Bogoo Biotechnology Co., Ltd., Shanghai, China) for 1 min and immersed in 1% ammonia to develop a blue color gamut, followed by dehydration using alcohol of gradient concentration and xylene clearing. The neutral gum-mounted sections were observed under a microscope (Olympus CX23, Olympus Corp., Japan).

### Cell stimulation

The TNBC cell lines (MDA-MB-231, MDA-MB-453, HCC-1937 and MDA-MB-468) and the human normal breast cell line (MCF-10A) (American type culture collection, Manassas, VA, USA) were cultured using medium containing 10% fetal bovine serum (Gibco Company, Grand Island, NY, USA), 89% Dulbecco’s minimum essential medium (DMEM) and 1% antibiotic in an incubator at 37°C with 5% CO_2_ (Thermo Fisher Scientific, Waltham, MA, USA). Cells at the logarithmic phase were detached using trypsin, seeded in a 6-well plate (1 × 10^5^ cells/well) and cultured for 24 h. Upon attaining 75% cell confluence, the cell transfection was initiated in strict accordance with the provided instructions of Lipofectamine 2000 (Invitrogen, Carlsbad, CA, USA). The plasmids of oe-negative control (NC) and oe-LINC00472 were purchased from Shanghai GenePharma Co., Ltd., (Shanghai, China).

### Reverse transcription quantitative polymerase chain reaction (RT-qPCR) for RNA quantitation

The total RNA of the aforementioned cells was extracted using the TRIzol reagent (15596026, Invitrogen, Carlsbad, CA, USA). RNA was reversely transcribed into complementary DNA (cDNA) according to the provided instructions of the PrimeScript RT reagent Kit (RR047A, Takara, Tokyo, Japan). The obtained cDNA was then subjected to RT-qPCR using the Fast SYBR Green PCR kit (Applied Biosystems, Oyster Bay, NY, USA) on the ABI 7500 RT-qPCR system (Applied Biosystems, Oyster Bay, NY, USA). Three duplicates were set for each well. The relative expression of genes was calculated based on the 2^-ΔΔCt^ method with glyceraldehyde-3-phosphate dehydrogenase (GAPDH) serving as the loading control. The primer sequences are shown in Table 2.

**Table 2:** Primer sequences for reverse transcription quantitative polymerase chain reaction.

**Target**	**Primer sequence**
LINC00472	F: 5'-CGGGATCCATGCGAGGCTGGGGCCGGTTG-3’
	R: 5'-CCGCTCGAGTTTAGACTCCAAATGGCTG-3’
GAPDH	F: 5'-GGTATCGTGGAAGGACTCATGAC-3’
	R: 5'-ATGCCAGTGAGCTTCCCGTTCAG-3’

Notes: GAPDH, glyceraldehyde-3-phosphate dehydrogenase; F, forward; R, reverse.

### Western blot analysis for protein measurement

Cells were collected by trypsin detachment and lysed using Radio-Immunoprecipitation Assay (RIPA) lysis buffer containing the protease inhibitors (Wuhan Boster Biological Technology Ltd., Wuhan, Hubei, China). The concentration of protein was determined using the bicinchoninic acid (BCA) kit (Wuhan Boster Biological Technology Ltd., Wuhan, Hubei, China). The protein was then separated by conducting sodium dodecyl sulphate-polyacrylamide gel electrophoresis and transferred onto a polyvinylidene difluoride (PVDF) membrane. A membrane blockade was performed for 1 h and incubated with the following primary rabbit anti-human antibodies to matrix metalloprotein-9 (MMP-9) (ab194316, 1 : 1000), N-cadherin (ab18203, 1 : 1000), Vimentin (ab137321, 1 : 500), E-cadherin (ab15148, 1 : 500), ERK1/2 (ab17942, 1 : 1000), phosphorylated ERK1/2 (ab214362, 1 : 500), MEK1/2 (ab178876, 1 : 5000, Abcam, Cambridge, UK), phosphorylated MEK1/2 (ab194754, 1 : 1000), and GAPDH (ab181602, 1 : 5000) at 4°C overnight. The aforementioned antibodies were provided by Abcam Inc. (Cambridge, UK). The protein bands were developed using the enhanced chemiluminescence reagent and the observations were documented using the SmartView Pro 2000 software (UVCI-2100, Major Science, USA). The Quantity One software analyzed the gray values of protein bands.

### Cell proliferation evaluation by 5-Ethynyl-2’-deoxyuridine (EdU) assay

Cells were seeded in a 24-well plate with three duplicates set for each group. Then, EdU (C10341-1, Guangzhou Ribo Biotechnology Co., Ltd., Guangzhou, Guangdong, China) was added to attain a final concentration of 10 μmol/L for incubation for 2 h. With removal of the medium, the cells were fixed using phosphate buffer saline (PBS) containing 4% paraformaldehyde for 15 min at room temperature, and incubated with PBS containing 0.5% Triton-100 for 20 min at room temperature. Then, every 100 μL of Apollo® 567 (Guangzhou Ribo Biotechnology Co., Ltd., Guangzhou, Guangdong, China) was supplemented into each well for a regimen of 30-min incubation at room temperature under conditions devoid of light. Subsequently, 1 × Hoechst 33342 was applied to cells for a regimen of 30-min staining. The mounted cells were observed under a fluorescence microscope (FM-600, Shanghai Puda Optical Instrument Co., Ltd., Shanghai, China) to count the number of positive cells (red).

### Cell migration evaluation by scratch test

Horizontal lines were marked at the bottom of the 6-well plate every 0.5 - 1 cm using a ruler and a marker pen. A minimum of five lines was made across each well. Cells were added into the 6-well plate at a density of 5 × 10^4^ cells/well and cultured in serum-free medium overnight. A sterile pipette (10 μL) was used to generate lines vertical to the horizontal lines at the bottom. The scratch distance was observed and measured under an optical microscope at 0 h and 48 h. Images were documented under an inverted microscope for observation on cell migration.

### Cell invasion evaluation by transwell assay

Matrigel (Becton, Dickinson and Company, Franklin Lake, NJ, USA) was paved on the upper side of the basement membrane in the Transwell chambers and allowed to polymerize into gel form at 37ºC for 30 min. The basement membrane was subjected to hydration before use. Cells were cultured using serum-free medium for 12 h, collected and re-suspended in serum-free medium (1 × 10^5^ cells/mL). The basolateral chambers were supplemented with medium containing 10% FBS. The cell suspension (100 μL) was added into the Transwell chambers and incubated at 37ºC for 24 h. Cells unable to invade through the Matrigel membrane were removed gently using a cotton swab. The remaining cells were fixed using 100% methanol and stained with 1% toluidine blue (Sigma-Aldrich Chemical Company, St Louis, MO, USA). The stained cells were observed under an inverted microscope (Carl Zeiss AG, Oberkochen, Germany). Five fields were selected on a random basis for cell counting.

### DNA methylation detection by Methylation-specific PCR (MSP)

The methylation level of the MCM6 promoter region was assessed using the DNA Methylation-Gold^TM^ kit (D5005, Zymo Research, Irvine, CA, USA). The primer sequences for MSP amplification were MCM6-MD (5’-ATTCGGATAAAATTTTTAGATTCGA-3’) and MCM6-MR (5’-TAAAAAAAACCCATCTACCTTTACG-3’) for the methylated reaction and MCM6-UD (5’-ATTTGGATAAAATTTTTAGATTTGA-3’) and MCM6-UR (5’-AAAAAAAACCCATCTACCTTTACAC-3’) for the unmethylated reaction. The purified DNA was supplemented with circulating tumor (CT) conversion reagent for denaturation and bisulfate conversion, followed by desulphurization and purification. The purified DNA was employed for subsequent PCR. The reaction products were separated by electrophoresis on agarose gel. Image analysis was performed using the gel electrophoresis imaging and image analysis system.

### Subcellular localization of LINC00472 by fluorescence *in situ* hybridization (FISH)

The subcellular localization of LINC00472 was detected by conducting FISH in compliance with the provided instructions of the Ribo^TM^ lncRNA FISH Probe Mix (Red) (Ribo Biotechnology Co., Ltd., Guangzhou, Guangdong, China). LINC00472 specific probe was generated according to the LINC00472 sequences. The coverslip was placed in a 6-well plate, where the TNBC cells were seeded and cultured overnight. Upon attaining 80% cell confluence, the coverslip was rinsed using PBS, fixed using 1 mL of 4% paraformaldehyde, and treated with proteinase K (2 μg/mL), glycine and the acetylation reagent. The cells were prehybridized using 250 μL of the prehybridization solution at 42ºC for 1 h. With removal of the prehybridization solution, 250 μL of hybridization solution (300 ng/mL) containing the LINC00472 specific probe was added for hybridization at 42ºC overnight. After 3 rinses with phosphate buffered saline with Tween-20 (PBST), the nucleus was stained using PBST-diluted 4',6-diamidino-2-phenylindole (DAPI) (1 : 800) for 5 min, followed by 3 rinses with PBST. Five visual fields were randomly selected and photographed under the fluorescence microscope (Olympus Corp., Tokyo, Japan).

### Subcellular fractionation location

The separation of the nuclear and cytosolic fractions was performed using the PARIS Kit (Life Technologies, Carlsbad, CA, USA) in strict accordance with the provided instructions [36].

### RNA-immunoprecipitation (RIP)

The binding of LINC00472 to DNMT1, DNMT3a and DNMT3b was detected using RIP kit (Millipore Corp, Billerica, MA, USA). Cells were collected and rinsed using pre-cold PBS. The supernatant was discarded. Then, the cells were lysed with an equivalent amount of the RIPA lysis buffer (P0013B, Beyotime Biotechnology Co., Ltd., Shanghai, China). The supernatant was collected. A portion of the supernatant was used as Input while the remaining portion was used for coprecipitation with an antibody to DNMT1 (1 : 100, ab13537), DNMT3a (1 : 100, ab2850) and DNMT3a (1 : 100, ab2851) at room temperature for 30 min. The human antibody to IgG (1 : 100, ab109489) was used as NC. The aforementioned antibodies were purchased from Abcam Inc., (Cambridge, UK). Every 50 μL beads were rinsed, re-suspended in 100 μL of the RIP Wash Buffer and incubated with 5 μg of the antibody. The bead-antibody complex was rinsed, re-suspended using 900 μL of the RIP Wash Buffer and incubated with 100 μL of cell extract at 4ºC overnight. The bead-protein complex was isolated on a magnetic base. RNA was extracted from the sample and Input after detachment using protease K for subsequent RT-qPCR.

### RNA pull-down assay

The TNBC cells were transfected with wild-type (WT) and mutant (MUT) biotinylated LINC00472 (50 nM) for 48 h, collected, rinsed using PBS and incubated with the specific cell lysis buffer (Ambion, Austin, TX, USA) for 10 min. Then 50 mL cell lysate was sub-packed. The lysate was incubated with M-280 streptavidin beads (Sigma-Aldrich Chemical Company, St Louis, MO, USA) at 4ºC for 3 h, which were pre-coated by RNase-free BSA and yeast tRNA (Sigma-Aldrich Chemical Company, St Louis, MO, USA). The beads were then rinsed using the cold lysis buffer 2 times, low-salt buffer 3 times and then once with high-salt buffer. The combined RNA was purified by a regimen of high-efficient RIPA lysis. The expression of DNMT1, DNMT3a and DNMT3b were determined by means of Western blot analysis.

### Chromatin immunoprecipitation (ChIP) assay

The ChIP kit (Millipore Corp, Billerica, MA, USA) was applied to analyze the binding of LINC00472 to DNMT1, DNMT3a and DNMT3b in the MCM6 promoter. Upon attaining 70% - 80% cell confluence, the cells were fixed using 1% formaldehyde at room temperature for 10 min to generate intracellular DNA-protein cross-links. Then, glycine was supplemented to terminate the fixation and the cells were then sonicated to produce chromatin fragments, followed by centrifugation (13000 rpm, 4ºC). The supernatant was sub-packed in 3 tubes and incubated with the RNA polymerase II antibody in the positive control, IgG antibody in the NC, and specific antibodies to DNMT1 (1 : 100, ab13537), DNMT3a (1 : 100, ab2850) and DNMT3a (1 : 100, ab2851) at 4ºC overnight. All antibodies were purchased from Abcam Inc. (Cambridge, UK). The endogenous DNA-protein complex was precipitated using the Protein Agarose/Sepharose. The supernatant was discarded through transient centrifugation. The non-specific complex was rinsed. The precipitate was isolated and the de-crosslinking was conducted at 65ºC overnight. The DNA fragments were extracted, purified and retrieved using phenol/chloroform. The enrichment of DNMT1, DNMT3a and DNMT3b in the MCM6 promoter was determined by means of RT-qPCR.

### Xenograft tumor in nude mice

A total of 36 specific pathogen free female athymic BALB/c nude mice (age: 5 weeks, weight: 18 - 20 g) were enrolled in the study. MDA-MB-231 cells (6 × 10^6^) stably transfected with oe-NC, oe-LINC00472 or co-transfected with oe-LINC00472 and oe-MCM6 were combined with Matrigel at a ratio of 1 : 1. The cell suspension (3 × 10^6^ cells/0.2 mL) was implanted subcutaneously into the right axilla (n = 12). The tumor growth was observed. The mouse weight and tumor size were measured on alternate days. After 5 weeks, the nude mice were euthanized by CO_2_ asphyxia to resect and weigh the tumors. The animal protocol and experiment procedures were in accordance with the recommendations of the International Guide for the Care and Use of Laboratory Animals.

### *In vivo* tumor metastasis assays

MDA-MB-231 cells (2 × 10^6^) stably transfected with oe-NC, oe-LINC00472 or co-transfected with oe-LINC00472 and oe-MCM6 were injected into the tail vein of female athymic BALB/c nude mice (n = 12). After 8 weeks, the nude mice were euthanized to resect the lungs. The number of metastatic nodules was counted and the morphological changes were observed by means of hematoxylin-eosin (HE) staining.

### HE staining

The lung tissues were sliced into sections, dewaxed, stained using hematoxylin (PT001, Shanghai Bogoo Biotechnology Co., Ltd., Shanghai, China) at room temperature for 10 min and rinsed under running water for 30 - 60 s. Then, 1% hydrochloric alcohol was supplemented for differentiation for 30 s. The sections were then stained using eosin (0001-H, Beijing Xinhualvyuan Technology Co., Ltd., Beijing, China) at room temperature for 1 min, dehydrated using alcohol of ascending concentration, and cleared with carbolic acid xylene I and II (GD-RY1215-12, Shanghai Guduo Biotechnology Co., Ltd., Shanghai, China). The neutral gum-mounted sections were finally observed under an optical microscope for morphological changes.

### Statistical analysis

The data were processed using the SPSS 21.0 statistical software (IBM, Armonk, NY, USA). Measurement data were expressed as mean ± standard deviation. Paired data following normal distribution and homogeneity between two groups were compared using the paired *t*-test, while the unpaired data were analyzed by the unpaired *t*-test. Comparisons among multiple groups were conducted based on one-way analysis of variance (ANOVA), followed by Tukey’s post hoc test. Data at different time points were compared by repeated measures ANOVA, followed by the Bonferroni post-hoc test. Pearson’s correlation coefficient was used to analyze the correlation between LINC00472 and Ki-67. A value of *p* < 0.05 was indicative of a statistically significant difference.
